# Patient diversity and author representation in clinical studies supporting the Surviving Sepsis Campaign guidelines for management of sepsis and septic shock 2021: a systematic review of citations

**DOI:** 10.1186/s12879-023-08745-4

**Published:** 2023-11-01

**Authors:** Lama Nazer, Aseel Abusara, Batoul Aloran, Tamas Szakmany, Hamza Nabulsi, Anton Petushkov, Marie-Laure Charpignon, Taghreed Ahmed, Marisa Cobanaj, Mohammad Elaibaid, Christian Lee, Chenyu Li, Donald Mlombwa, Sulaiman Moukheiber, Anupol Panitchote, Rachael Parke, Skyler Shapiro, Naira Link Woite, Leo Anthony Celi

**Affiliations:** 1 King Hussein Cancer Center, Amman, Jordan; 2 Cardiff University, Cardiff, Wales, UK; 3 University of Jordan, Amman, Jordan; 4 University of Michigan, Ann Arbor, USA; 5 Massachusetts Institute of Technology, Massachusetts, USA; 6 Omdurman Military Hospital, Khartoum, Sudan; 7 OncoRay, Dresden, Germany; 8 Police’s Hospital, Khartoum, Sudan; 9 Troy High School, California, USA; 10 University of Pittsburgh School of Medicine, Pittsburgh, USA; 11 Zomba Central Hospital, Zomba, Malawi; 12 Worcester Polytechnic Institute, Massachusetts, USA; 13 Khon Kaen University, Khon Kaen, Thailand; 14 Auckland City Hospital, Auckland, New Zealand; 15 Cornell University, New York, USA; 16 Harvard T.H. Chan School of Public Health, Massachusetts, USA; 17 Beth Israel Deaconess Medical Center, Massachusetts, Boston USA

**Keywords:** Sepsis, Guidelines, Diversity, Geographic mapping, Racial groups, Sex, Gender

## Abstract

**Background:**

The generalizability of the Surviving Sepsis Campaign (SSC) guidelines to various patient populations and hospital settings has been debated. A quantitative assessment of the diversity and representation in the clinical evidence supporting the guidelines would help evaluate the generalizability of the recommendations and identify strategic research goals and priorities. In this study, we evaluated the diversity of patients in the original studies, in terms of sex, race/ethnicity, and geographical location. We also assessed diversity in sex and geographical representation among study first and last authors.

**Methods:**

All clinical studies cited in support of the 2021 SSC adult guideline recommendations were identified. Original clinical studies were included, while editorials, reviews, non-clinical studies, and meta-analyses were excluded. For eligible studies, we recorded the proportion of male patients, percentage of each represented racial/ethnic subgroup (when available), and countries in which they were conducted. We also recorded the sex and location of the first and last authors. The World Bank classification was used to categorize countries.

**Results:**

The SSC guidelines included six sections, with 85 recommendations based on 351 clinical studies. The proportion of male patients ranged from 47 to 62%. Most studies did not report the racial/ ethnic distribution of the included patients; when they did so, most were White patients (68–77%). Most studies were conducted in high-income countries (77–99%), which included Europe/Central Asia (33–66%) and North America (36–55%). Moreover, most first/last authors were males (55–93%) and from high-income countries (77–99%).

**Conclusions:**

To enhance the generalizability of the SCC guidelines, stakeholders should define strategies to enhance the diversity and representation in clinical studies. Though there was reasonable representation in sex among patients included in clinical studies, the evidence did not reflect diversity in the race/ethnicity and geographical locations. There was also lack of diversity among the first and last authors contributing to the evidence.

## Background

Sepsis is a life-threatening medical emergency associated with an annual incidence of about 49 million cases worldwide and contributing to 11 million deaths [1]. The incidence and mortality of sepsis vary among regions, with the highest health-related burden reported in sub-Saharan Africa and other low- and middle-income countries, although data from these settings remain scarce and lack granularity [1]. A large study evaluating the epidemiology of sepsis across 730 intensive care units globally demonstrated that sepsis mortality might increase by two-fold depending on the geographical location [2].

The Surviving Sepsis Campaign (SSC) is an international initiative that was introduced in 2002 at the European Society of Intensive Care Medicine annual meeting in Barcelona, with the “Barcelona Declaration” [3]. It was formed by three professional organizations: the European Society of Intensive Care Medicine, the Society of Critical Care Medicine, and the International Sepsis Forum [3]. The first SSC evidence-based guidelines were published in 2004, followed by updates almost every 4 years. The guidelines were meant to be relevant to the entire world, and therefore, there has been emphasis over the years to have the working panel represent the diverse populations of patients and providers it serves, with diversity in race and gender, as well as national, geographical, and income-settings [3]. For example, the proportion of women increased from 10 to 28%, and the geographical representation outside of North America and Europe increased from 5 to 25% in the 2004 and 2021 guidelines committees, respectively [3]. In addition, to further optimize the recommendations, the SSC established the SSC Research Committee that identified a list of research priorities in various areas related to sepsis [4].

Despite the attempt to increase the diversity of the working group and the quality of available evidence that supports the sepsis guidelines, significant debate and concerns have been raised. Low- and middle-income countries have struggled to implement the proposed diagnostic pathways, with the simplified risk assessment tools repeatedly shown to be deficient in these settings [5–7]. Furthermore, some of the therapeutic interventions ubiquitous in high-income countries, such as fluids for resuscitation, have been shown to instead increase mortality among patients in sub-Saharan Africa [8–10]. A major contributing factor is related to the patient representation within the evidence utilized to support the guidelines.

Diversity and inclusion is essential not only in recruiting the patient populations underlying clinical studies, but also in building the teams undertaking them. In a large-scale examination of over 6 million papers across the medical sciences since 2000, Yang et al. demonstrated that gender-diverse teams tend to produce more novel and higher-impact scientific ideas [11]. Furthermore, diverse author groups have been shown to collaborate more effectively and to achieve scientific output of significantly higher quality [12].

To be able to identify specific strategies and research priorities to enhance the generalizability of the sepsis guidelines, it is critical to have a quantitative assessment of the representation in the evidence supporting such guidelines. We hypothesized that there is insufficient diversity in the patients enrolled in the studies, as well as among authors of the available evidence. Therefore, we conducted this study that aimed to investigate the extent of diversity among patients and authors who contributed to evidence supporting the most recent SSC international guidelines for management of sepsis and septic shock in adults, published in 2021. First, we determined the distribution of patient populations in the original studies underpinning the recommendations, in terms of their sex, race/ethnicity, and country of origin. Second, we examined the composition of the research teams leading those studies by evaluating sex and geographical representation amongst first and last authors.

## Methods

The recommendations in the 2021 SSC international guidelines for management of sepsis and septic shock in adults were divided into the following sections, based on the classification provided in the guidelines: (1) Screening and early treatment (recommendations 1–10); (2) Infection (recommendations 11–31); (3) Hemodynamic management (recommendations 32–45); (4) Ventilation (recommendations 46–57); (5) Additional therapies (recommendations 58–73); (6) Long-term outcomes and goals of care (recommendations 74–93) [13]. Recommendations that were listed but did not include any specific guidance were excluded.

For each section, we identified all references cited in support of the specific recommendations, listed under the rationale sections. Eligible references included original clinical studies (interventional, observational, and surveys). We excluded non-clinical studies, reviews, editorials, other guidelines, and meta-analyses. In addition, references written in another language than English and that did not have an English translation of the full publication were excluded. Identifying the cited references and assessing their eligibility was performed by one of the investigators and then reviewed by another investigator. A list of the inclusion and exclusion criteria was provided to the reviewers to guide the assessment. Any discrepancies were discussed between the reviewers and the primary investigator.

For each study, we extracted the sociodemographic composition of the underlying patient cohort. Specifically, we recorded the total sample size and the number of male patients. We also recorded whether race/ethnicity was described and the number of patients within each reported racial/ethnic group. Although race and ethnicity reflect different aspects of identity, we combined both in our data collection and analysis since study authors tend to use them interchangeably when describing the sociodemographic characteristics of participants. We used the racial/ethnic categories and definitions of the National Institute of Health (NIH) for reporting, which included: (1) American Indian or Alaska Native; (2) Asian; (3) Black or African American; (4) Hispanic or Latino; (5) Native Hawaiian or other Pacific Islander; (6) White [14]. For each study, patient-related data were extracted from the results section of the manuscript.

Additionally, we determined the country(ies) in which the study was conducted. The World Bank classification was used to classify countries by income group: low, lower-middle, upper-middle, and high-income. [15] The countries were also classified into the following regions, based on the World Bank classification: East Asia and Pacific, Europe and Central Asia, Latin America and Caribbean, Middle East and North Africa, North America, South Asia, and Sub-Saharan Africa. [15]. The characteristics of the patient cohorts as well as the countries in which the studies were conducted were extracted by two investigators who recorded the data independently and then compared their results. Each feature was clearly defined to ensure consistency among the reviewers. When there was a discrepancy in the extracted data between the reviewers, a third reviewer evaluated the results and, if needed, discussed them with the two other investigators and the primary investigator to reach full consensus.

To examine authorship, we evaluated the first author and last author for each study. The first author was chosen since that reflects the person who contributed the most and receives most of the credit while the last author is assumed to be the senior more experienced person and is the driving force, intellectually and possibly financially, for the study [16]. To evaluate sex and country representation among the first and last authors, we identified the PubMed Unique Identifiers (PMIDs) for all eligible studies and uploaded them to Dimensions AI (www.dimensions.ai). Dimensions AI is an application-programming interface (API) that can be used to retrieve scientometric data, including the country of an author based on the institutional affiliation referred to in the specific citation. The Dimensions AI query yielded the list of authors’ names and their institutional affiliations, from which we derived the corresponding geographical locations. Subsequently, we used Genderize (www.genderize.io) to determine the sex of the first and last authors of each study. Genderize is an API that is used to predict the most likely sex/ gender of a person based on their first name and classifies them based on the vector of probabilities corresponding to female, male, or unknown. For names whose sex was classified by Genderize as “unknown” and for geographical information that could not be retrieved from Dimensions AI, one of the investigators annotated the missing data points manually. When the sex of the author could not be determined through manual annotation (e.g., first name listed as initials or unisex names), we assigned the label as “unknown”. Subsequently, about 20% of the extracted data underwent a random check by the primary investigator The assignment of country categories (i.e., income group and region) to first and last authors was performed using the same World Bank classification as that used for the countries in which the studies were conducted [15].

### Statistical analysis

Descriptive statistics were used to report the sociodemographic characteristics of patients included in the studies as well as those of the first and last authors. Clinical studies included as citations under more than one section of the guidelines were analyzed under each; however, if the study was cited more than once under the same section of the recommendations, the duplicates were removed to derive the sociodemographic characteristics of the corresponding study cohort.

First and last authors who published multiple unique studies under the same section contributed to the assessment of gender diversity and country representation for each distinct paper that they appeared on (i.e., they were accounted for based on the number of times they appeared in the included citations).

For eligible studies that included family, caregivers, healthcare members as participants (mostly survey studies), the characteristics of the participants were combined and analyzed with those of patients as they were deemed likely to reflect characteristics and geographical locations like those of the patients they represent.

## Results

**Fig. 1:**
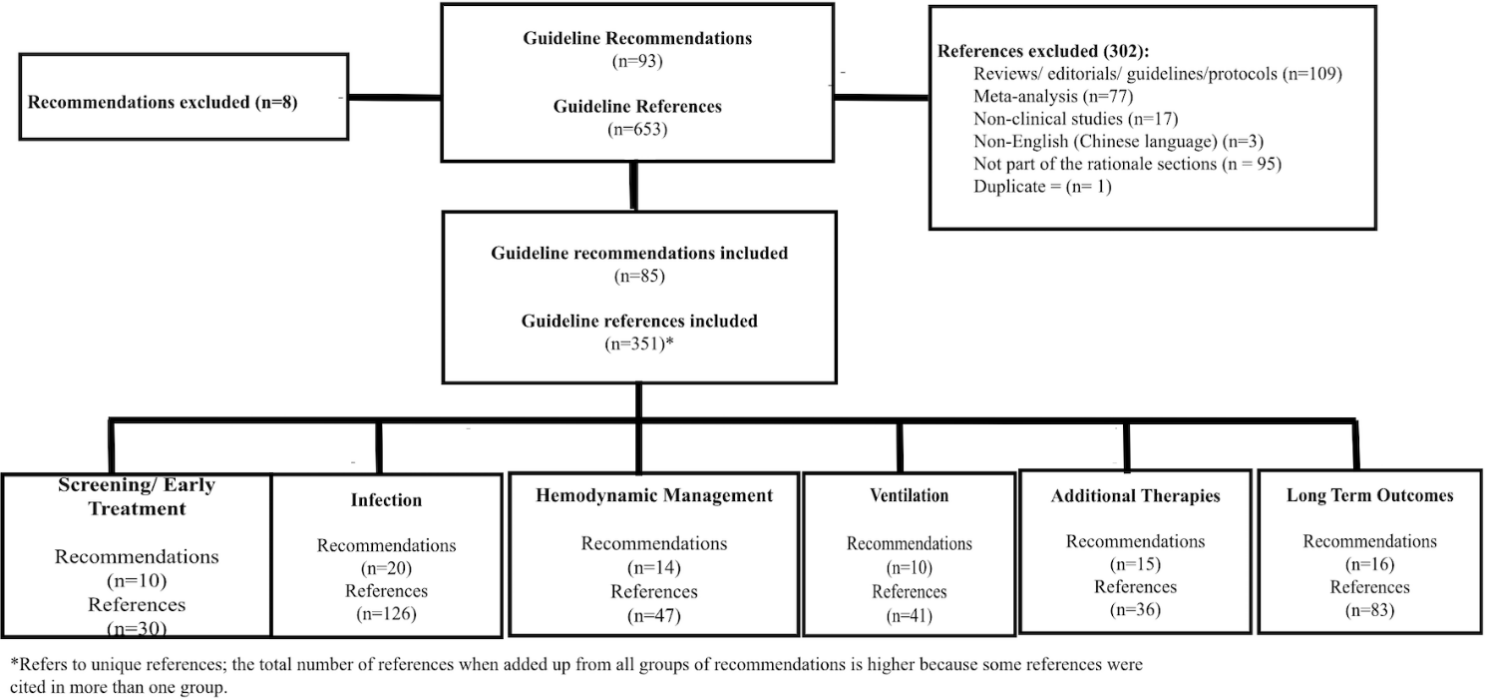
Flow diagram for inclusion and exclusion of references that support the Surviving Sepsis Campaign guideline recommendations

The 2021 SSC international guidelines for management of sepsis and septic shock in adults comprised a total of 93 recommendations and 653 references. Among those, we included 85 recommendations and 351 references to original clinical studies that supported the rationale for the guideline recommendations. Eight recommendations did not include specific guidance and were therefore excluded. Figure 1 outlines the references included and excluded from the analysis and details those that were included under each section of the recommendations.

The sociodemographic distribution of patients included in the studies that supported each section of recommendations is presented in Table 1, by sex and race/ethnicity. The proportion of male patients ranged from 47% in studies supporting the recommendations related to long-term outcomes/goals of therapy to 62% in those supporting recommendations for ventilation. Most studies did not report patient race/ethnicity. Among those that provided racial/ethnic characteristics (14–36%), most patients were reported to be White (68–77%), followed by Black/African American (14–19%), and Hispanic/Latino (5–15%).

**Table 1:** Sex and race/ethnic representation in the original studies supporting the 2021 Surviving Sepsis Campaign guidelines

Recommendations	Studies (n)	Sample size (n)	Males^1^ n (%)	Reported race^2^ n (%)	White^3^ n (%)	Black^3^ n (%)	Hispanic^3^ n (%)	Others^3^ n (%)
**Screening & Early Treatment**	30	2,741,771	1,300,889 (48)	5 (17)	793,221 (68)	193,612 (17)	94,769 (9)	81,421 (7)
**Infection**	126	779,053	424,672 (61)	21 (17)	134,091 (73)	28,320 (16)	2,861 (12)	17,731 (10)
**Hemodynamic Management**	47	68,939	37,531 (56)	10 (21)	32,623 (74)	3,508 (14)	3 (15)	4,049 (17)
**Ventilation**	41	28,683	13,732 (62)	6 (15)	2,178 (74)	390 (16)	205 (9)	57 (4)
**Additional Therapies**	36	232,790	123,081 (54)	5 (14)	1057 (77)	181 (19)	30 (5)	40 (4)
**Long Term Outcomes/** **Goals of Care**	83	2,508,901	1,183,748 (47)	30 (36)	838,323 (71)	209,136 (17)	87,554 (8)	42,648 (4)

^1^Some studies may not have reported the sex; the proportion of males reflects the proportion from the studies that reported sex

^2^Refers to the number of studies that had race reported in the patient characteristics

^3^Refers to the proportion based on the studies that reported each of the listed race/ethnicity; total may not add up to 100% given that there is inconsistency in the race/ethnicity reported by each study

**Fig. 2:**
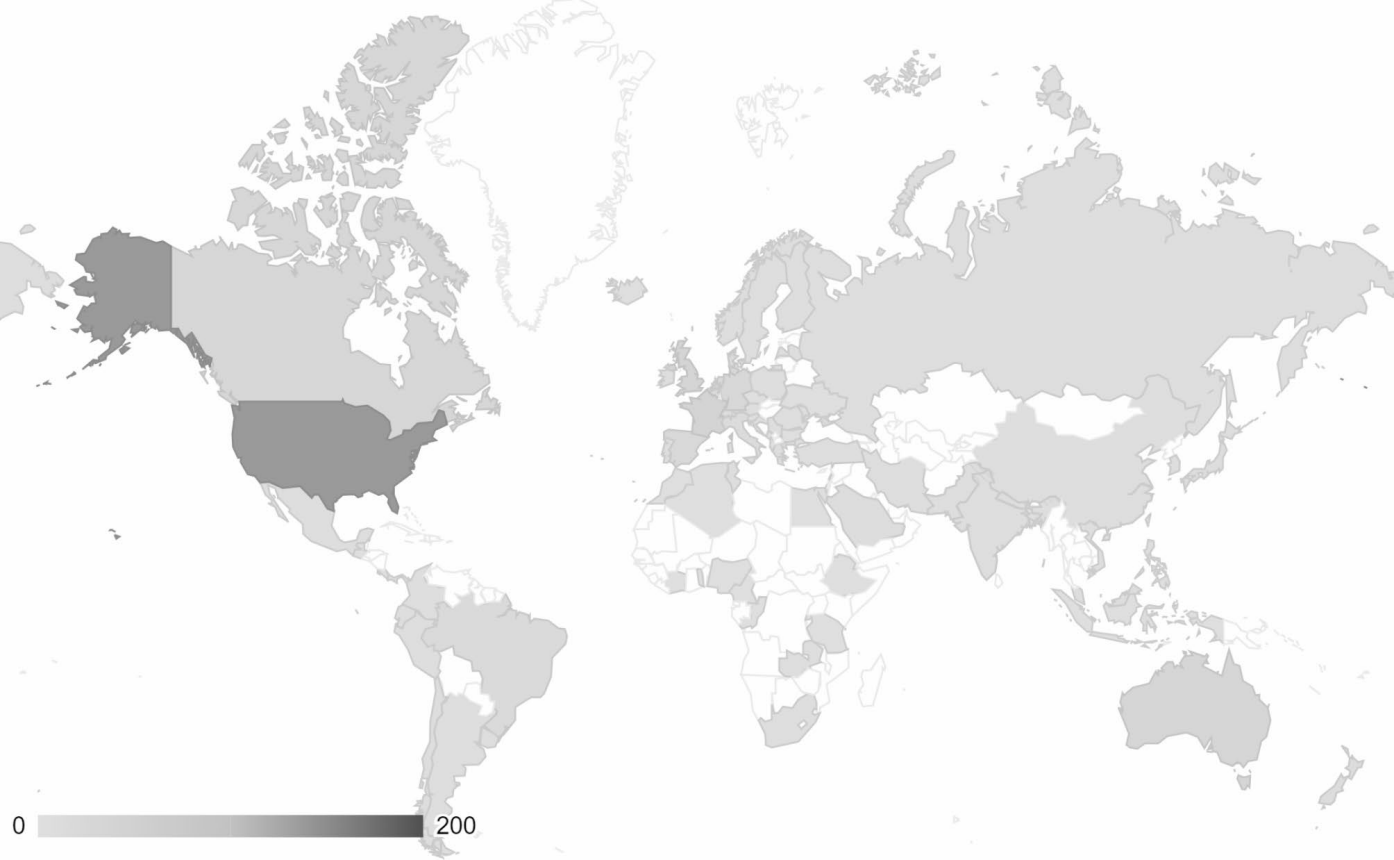
Geographic representation among clinical studies supporting the Surviving Sepsis Campaign guideline recommendations

The geographical representation of patient populations in studies supporting each section of recommendations in the SSC guidelines is outlined in Tables 2 and 3, which show the distribution of countries in which the studies were conducted, by income level and region, respectively. Most clinical studies providing evidence in support of the guidelines were conducted in high-income countries (77-99%). Most studies included patients from Europe/Central Asia (33–66%) and North America (36–55%). Across all sections of the recommendations, representation from other regions was minimal (Fig. 2).

**Table 2:** Geographical representation in the original studies supporting the 2021 Surviving Sepsis Campaign guidelines based on the World Bank classification for income

Recommendation	Studies (n)	High n (%)	Upper Middle n (%)	Lower-Middle n (%)	Low n (%)
**Screening & Early Treatment**	30*	23 (77)	5 (17)	3 (10)	4 (13)
**Infection**	126*	114 (90)	16 (13)	10 (8)	8 (6)
**Hemodynamic Management**	47*	42 (89)	3 (6)	4 (9)	2 (4)
**Ventilation**	41*	39 (95)	9 (22)	0 (0)	0 (0)
**Additional Therapies**	36*	33 (92)	3 (8)	2 (6)	0 (0)
**Long Term Outcomes/** **Goals of Care**	83	82 (99)	1 (1)	0 (0)	0 (0)

*Total number of studies is higher than that of the total number of studies from each classification group since some studies were conducted in more than one country with different income classifications

**Table 3:** Geographical representation in the original studies supporting the 2021 Surviving Sepsis Campaign guidelines based on World Bank classification for regions

Recommendation	Studies (n)	East Asia Pacific n (%)	Europe Central Asia n (%)	Latin America Caribbean n (%)	Middle East North Africa n (%)	North America n (%)	South Asia n (%)	Sub-Saharan Africa n (%)
**Screening & Early Treatment**	30*	2 (7)	10 (33)	4 (13)	1 (3)	14 (47)	0 (0)	4 (13)
**Infection**	126*	25 (20)	55 (44)	12 (10)	9 (7)	54 (43)	3 (2)	8 (6)
**Hemodynamic Management**	47*	9 (19)	16 (34)	1 (2)	3 (6)	25 (53)	0 (0)	1 (2)
**Ventilation**	41*	8 (20)	27 (66)	10 (24)	5 (12)	19 (46)	0 (0)	1 (2)
**Additional Therapies**	36*	3 (8)	19 (53)	2 (6)	2 (6)	13 (36)	2 (6)	0 (0)
**Long Term Outcomes/** **Goals of Care**	83	7 (8)	31 (37)	0 (0)	0 (0)	46 (55)	0 (0)	0 (0)

*Total number of studies is higher than that of the total number of studies from each classification group since some studies were conducted in more than one region

The sociodemographic characteristics of the first and last authors of citations included in our analysis are shown in Table 4. Among first authors, the proportion of males ranged between 55% and 88%, while that among last authors ranged between 55% and 93%. Across all groups of recommendations, most first and last authors were affiliated with institutions located in high-income countries (77–99%). Specifically, the most commonly represented regions were Europe/Central Asia (27–51%) and North America (31–55%).

**Table 4:** Sex and country representation in the authorship of evidence supporting the 2021 SSC recommendations

Recommendation	First Author Sex: n (%)	First Author Country Income* n (%)	First Author Country Region** n (%)	Last Author Sex, n (%)	Last Author Country Income, n (%)	Last Author Country Region, n (%)
Screening and Early Treatment (n = 30)	Males: 24 (80%) Females: 5 (17%) Unknown: 1 (3%)	High: 23 (77%) UM: 3 (10%) LM: 1 (3%) Low: 3 (10%)	EAP: 1 (3%) ECA: 8 (27%) LAC: 5 (17%) MENA: 0 (0%) NA: 13 (43%) SA: 0 (0%) SSA: 3 (10%)	Males: 23 (77%) Females: 7 (23%)	High: 26 (87%) UM: 3 (10%) LM: 0 (0%) Low: 1 (3%)	EAP: 0 (0%) ECA: 12 (40%) MENA: 0 (0%) NA: 13 (43%) SA: 0 (0%) SSA: 1 (3%) LAC: 4 (14%)
Infection (n = 126)	Males: 84 (67%) Females: 29 (23%) Unknown: 13 (10%)	High: 116 (92%) UM: 6 (5%) LM: 2 (2%) Low: 2 (2%)	EAP: 22 (17%) ECA: 47 (37%) LAC: 5 (4%) MENA: 4 (3%) NA: 46 (37%) SA: 0 (0%) SSA: 2 (2%)	Males: 94 (75%) Females: 20 (16%) Unknown: 12 (9%)	High: 118 (94%) UM: 4 (3%) LM: 1 (1%) Low: 0 (0%) Unknown: 3 (2%)	EAP: 17 (14%) ECA: 47 (37%) LAC: 4 (3%) MENA: 4 (3%) NA: 51 (41%) SA: 0 (0%) SSA: 0 (0%) Unknown: 3 (2%)
Hemodynamic Management (n = 47)	Males :39 (83%) Females: 7 (15%) Unknown: 1 (2%)	High: 41 (87%) UM: 2 (4%) LM: 3 (7%) Low: 1 (2%)	EAP: 5 (11%) ECA: 13 (28%) LAC: 2 (4%) MENA: 1 (2%) NA: 23 (49%) SA: 1 (2%) SSA: 2 (4%)	Males: 34 (72%) Females: 10 (21%) Unknown: 3 (7%)	High: 42 (89%) UM: 2 (4%) LM: 3 (7%) Low: 0 (0%)	EAP: 5 (11%) ECA: 13 (28%) LAC: 2 (4%) MENA: 1 (2%) NA: 24 (51%) SA: 1 (2%) SSA: 1 (2%)
Ventilation (n = 41)	Males: 36 (88%) Females: 5 (12%)	High: 36 (88%) UM: 5 (12%) LM: 0 (0%) Low: 0 (0%)	EAP: 1 (2%) ECA: 17 (42%) LAC: 5 (12%) MENA: 0 (0%) NA: 18 (44%) SA: 0 (0%) SSA: 0 (0%)	Males: 38 (93%) Females: 2 (5%) Unknown: 1 (2%)	High: 36 (88%) UM: 4 (10%) LM: 0 (0%) Low: 0 (0%) Unknown: 1 (2%)	EAP: 0 (0%) ECA: 21 (51%) LAC: 4 (10%) MENA: 0 (0%) NA: 15 (37%) SA: 0 (0%) SSA: 0 (0%) Unknown: 1 (2%)
Additional therapies (n = 36)	Males: 29 (81%) Females: 7 (19%)	High: 33 (92%) UM: 1 (3%) LM: 2 (5%) Low: 0 (0%)	EAP: 4 (11%) ECA: 17 (47%) MENA: 1 (3%) NA: 11 (30%) SA: 2 (6%) SSA: 0 (0%) LAC: 1 (3%)	Males: 29 (80%) Females: 6 (17%) Unknown: 1 (3%)	High: 33 (92%) UM: 1 (3%) LM: 2 (5%) Low: 0 (0%)	EAP: 2 (6%) ECA: 18 (50%) MENA: 1 (3%) NA: 12 (33%) SA: 2 (6%) SSA: 0 (0%) LAC: 1 (3%)
Long-term Outcomes/ Goals of Care (n = 83)	Males: 46 (55%) Females: 35 (42%) Unknown: 2 (3%)	High: 82 (99%) UM: 1 (1%) LM: 0 (0%) Low: 0 (0%)	EAP: 7 (8%) ECA: 30 (36%) MENA: 0 (0%) LAC: 0 (0%) NA: 46 (55%) SA: 0 (0%) SSA: 0 (0%)	Males: 46 (55%) Females: 35 (42%) Unknown: 2 (3%)	High: 82 (99%) UM: 1 (1%) LM: 0 (0%) Low: 0 (0%)	EAP: 7 (8%) ECA: 30 (36%) MENA: 0 (0%) LAC: 0 (0%) NA: 46 (55%) SA: 0 (0%) SSA: 0 (0%)

*Based on the World Bank classification of countries by income to the following: High, Upper Middle (UP), Lower-Middle (LM), and Low

**Based on the World Bank classification of countries by region to the following: East Asia Pacific (EAP), Europe and Central Asia (ECA), Latin America Caribbean (LAC) Middle East and North Africa (MENA), North America (NA), South Asia (SA), and Sub-Saharan Africa (SSA).

## Discussion

In this study, we assessed diversity and representation among both patients and authors of the clinical studies supporting recommendations in the 2021 SSC international guidelines for management of sepsis and septic shock in adult patients. To our knowledge, this is the first study providing an in-depth assessment of the representation in the evidence used to derive international practice guidelines. Although the overall patient distribution was relatively balanced with respect to sex, most studies were composed of White participants from high-income regions. Similarly, we found that most study first and last authors were males from high-income countries. Though our findings likely reflect the overall lack of diversity in available clinical evidence rather than selection bias, they highlight an important limitation of most international practice guidelines.

Potential harms resulting from a lack of diversity in the evidence supporting the SSC guidelines were previously raised by studies that evaluated guidelines about early resuscitation with intravenous fluid boluses and vasopressors in resource-limited countries [8–10]. Andrews et al. conducted a randomized controlled study among Zambian adults to evaluate a resuscitation protocol with administration of intravenous fluids, vasopressors, and blood transfusions, early after presentation to the emergency department [8]. Although the sepsis protocol resulted in seemingly positive measures – consisting in greater fluid administration, vasopressor use, and lactate clearance, patients who were randomized to early resuscitation had more frequent worsening of their hypoxemia and tachypnea and higher mortality than patients who received usual care.

Despite concerns raised about a decade ago regarding the lack of diversity and inclusion of patient populations from countries located in resource-limited regions with a high prevalence of sepsis, the available guidelines are still derived using clinical evidence originating mainly from high-income countries. A study that evaluated the recommendations for fluid management in ten widely used international sepsis guidelines, including those from the SSC, reported a lack of high-quality evidence from sub-Saharan Africa [17]. The risk is for such findings to yield disparate recommendations, lack of specificity in sepsis identification, and protocols mostly unattainable in low-income countries with limited resources.

Diversity in the sex and race/ethnicity of the study participants is also important. Clinical studies reported differences in the incidence and outcomes of sepsis between males and females [18]. In addition, several studies demonstrated the differential impact of race, as well as other social determinants of health, on the incidence, severity, and outcomes of sepsis [19, 20]. Such examples underscore that guidelines generated using clinical evidence lacking sufficient diversity may not necessarily yield the same anticipated outcomes in less represented patient groups.

In a recent study that evaluated the quality of evidence supporting the 2017 SSC recommendations, Rello et al. concluded that most recommendations were informed by indirect evidence and non-systematic observations [21]. To address this limitation, the authors proposed Delphi-like approaches or multi-criteria decision analysis to guide recommendations [21]. Diversity among participants in such studies is a key factor to enhance the diversity of the generated evidence.

Interestingly, our examination of the clinical studies underpinning the SSC guidelines revealed not only the under-representation of non-White racial/ethnic groups, but also the lack of reporting of such sociodemographic factors in most studies. Our findings corroborate those of McCambridge et al., who found that 12 out of 14 studies published in the Journal of Physiotherapy in 2020 did not report any information about race/ethnicity [22]. Without sufficient data about race/ethnicity and other social determinants of health, and without the distribution of patient outcomes stratified by subpopulation/sub-group, the clinical generalizability and applicability of the study findings would remain unknown.

Although diversity and representation in clinical research are desirable and crucial to derive guidelines that are generalizable to various patient populations and settings, extensive national and international efforts are necessary to achieve such a goal. Recently, the Food and Drug Administration released guidance towards researchers and pharmaceutical companies, providing recommendations for developing a Race and Ethnicity Diversity Plan to enroll adequate numbers of participants from under-represented racial and ethnic subpopulations in the United States [23]. While this is certainly a critical step to enhance diversity in clinical trials, the barriers to access and enrollment of under-represented populations in clinical studies should also be better understood [24]. In addition, coordinated investments are needed to expand research capacity in developing countries in a structured and sustainable manner [25, 26]. Concurrently, journal editors, reviewers, and publishers also play an important role by ensuring the comprehensive reporting and analysis of patient sociodemographic characteristics in published research. The Sex and Gender Equity in Research guidelines that were developed to guide authors in reporting and interpreting sex- and gender-related information in their studies could also be used by editors and reviewers, as gatekeepers of science, to integrate assessment of sex and gender into published manuscripts [27].

Abdel-Rahman et al. proposed a benchmarking strategy to evaluate racial/ethnic representation in clinical research, with the intent of increasing accountability for diversity and inclusion in clinical studies [28]. Specifically, the authors proposed a “diversity index” that consolidates individual subgroup statistics into a single value that is further transformed into a “representation quotient” (RQ). Furthermore, Gallifant et al. developed a “diversity factor” for measuring a journal’s contribution to the research landscape, focusing on diversity, equity, inclusion, and impact of the study population [29]. It is intended to remind journals and authors to assess how data reaches the manuscript and whether they consider all perspectives, not just those available. We propose developing a similar approach to systematically grade the guidelines based on the extent of sex, gender, racial/ethnic, and geographical representation of the clinical evidence informing specific recommendations. A “diversity score/grade” would resemble the GRADE system used to describe the quality of the evidence and strength of guideline recommendations [30]. Such a grading mechanism requires extensive efforts from a diverse group of investigators to develop and validate but would certainly provide clinicians with a quantitative measure of the generalizability of the recommendations in place and guide researchers in identifying areas of greatest need for further investigation to support evidence-based practice.

Despite the demonstrated value of authorship diversity, under-representation of women in research remains prevalent, particularly at senior levels. In a study that evaluated over 18,000 critical care research publications in 40 journals between 2008 and 2018, women comprised only 30% of first authors and less than 20% of senior authors – with minimal change over the considered ten-year period [31]. To achieve diversity among authors, several strategies have been suggested, such as implementing policies for diversity and inclusion, emphasizing mentorship of under-represented research groups, documenting diversity in publications, as well as training for diversity, equity, and anti-racism [32].

This project was a collaboration between individuals from diverse backgrounds in terms of disciplines, practice settings, clinical and research experiences, geographical locations, as well as sex and age groups. The authors included critical care physicians, pharmacists, nurses, respiratory therapist, engineers, data scientists, and students. The group represented various geographical areas which included: Africa, Asia, North America, Europe, Middle East, and Oceania. Our findings, based on a sizable sample of clinical studies, highlight persisting issues about the lack of inclusivity in terms of both study participants and authors of the evidence used to derive clinical guidelines. Nevertheless, the study has several limitations. First, we relied on the patient characteristics publicly reported in the considered clinical studies and did not contact the authors to request additional information that may have been collected but not necessarily reported (e.g., race/ethnicity of patients enrolled in a trial). Moreover, we limited our examination of patient sociodemographics to sex, race/ethnicity, and geographical location. We acknowledge that other social determinants of health, such as age, socioeconomic status, and educational attainment, may also affect the generalizability of clinical evidence and guidelines across patient populations. However, such variables either varied significantly in how they were reported across studies (e.g., age as a categorical variable with differing levels of granularity across studies) or were not reported in most studies (e.g., socioeconomic status). In regard to authorship, we evaluated the geographical and sex diversity for the first and last authors of the included studies; it is possible that a full bibliometric analysis of all contributing authors would have revealed more diverse geographical contributions; however, we believe that for our purposes, this could be captured in the patient level data, which included all the countries in which the study was conducted. In addition, there is the potential for misclassification bias as a result of inaccuracies in the sex and geographic location of authors retrieved from Genderize and Dimensions, respectively. However, the manual review of such data is not feasible, and we expect that the inaccuracies are minimal, given that such an approach has been used by other studies and that we did a random check for the results [33, 34]. Finally, our assessment for authorship did not include diversity in professional backgrounds, practice settings, and levels of seniority in research; yet these additional dimensions would provide a more holistic assessment of diversity.

## Conclusions

The findings of our study serve as a call to action. To ensure the relevance of sepsis guidelines and others for clinical practice, we need to identify and implement strategies to enhance the diversity and global representation in patients, healthcare settings, and authors contributing to the evidence supporting clinical practice guidelines. We recommend that stakeholders, including clinicians, academic researchers, journal reviewers and editors, patient advocacy groups, and health equity scholars – across institutions and across countries – jointly define a plan to mitigate the issue of under-representation.

## Data Availability

The data extracted and the dataset used for the current study is available from the corresponding author on reasonable request.

## Abbreviations

SSC: Surviving Sepsis Campaign
PMIDs: PubMed Unique Identifiers
